# Dexamethasone protected human glioblastoma U87MG cells from temozolomide induced apoptosis by maintaining Bax:Bcl-2 ratio and preventing proteolytic activities

**DOI:** 10.1186/1476-4598-3-36

**Published:** 2004-12-08

**Authors:** Arabinda Das, Naren L Banik, Sunil J Patel, Swapan K Ray

**Affiliations:** 1 Department of Neurology, Medical University of South Carolina, Charleston, USA; 2 Department of Neurosurgery, Medical University of South Carolina, Charleston, USA

**Keywords:** Apoptosis, Dexamethasone, Glioblastoma, Proteolysis, Temozolomide

## Abstract

**Background:**

Glioblastoma is the deadliest and most prevalent brain tumor. Dexamethasone (DXM) is a commonly used steroid for treating glioblastoma patients for alleviation of vasogenic edema and pain prior to treatment with chemotherapeutic drugs. Temozolomide (TMZ), an alkylating agent, has recently been introduced in clinical trials for treating glioblastoma. Here, we evaluated the modulatory effect of DXM on TMZ induced apoptosis in human glioblastoma U87MG cells.

**Results:**

Freshly grown cells were treated with different doses of DXM or TMZ for 6 h followed by incubation in a drug-free medium for 48 h. Wright staining and ApopTag assay showed no apoptosis in cells treated with 40 μM DXM but considerable amounts of apoptosis in cells treated with 100 μM TMZ. Apoptosis in TMZ treated cells was associated with an increase in intracellular free [Ca^2+^], as determined by fura-2 assay. Western blot analyses showed alternations in the levels of Bax (pro-apoptotic) and Bcl-2 (anti-apoptotic) proteins resulting in increased Bax:Bcl-2 ratio in TMZ treated cells. Western blot analyses also detected overexpression of calpain and caspase-3, which cleaved 270 kD α-spectrin at specific sites for generation of 145 and 120 kD spectrin break down products (SBDPs), respectively. However, 1-h pretreatment of cells with 40 μM DXM dramatically decreased TMZ induced apoptosis, decreasing Bax:Bcl-2 ratio and SBDPs.

**Conclusion:**

Our results revealed an antagonistic effect of DXM on TMZ induced apoptosis in human glioblastoma U87MG cells, implying that treatment of glioblastoma patients with DXM prior to chemotherapy with TMZ might result in an undesirable clinical outcome.

## Background

Glioblastoma patients usually receive steroids for alleviation of vasogenic edema and pain prior to treatment with chemotherapeutic drugs. Steroids, however, may modulate the sensitivity of tumor cells to chemotherapeutic drugs. Dexamethasone (DXM), a synthetic glucocorticoid, is commonly used to reduce inflammation and pain associated with glioblastoma [1]. However, DXM has been reported to make human glioblastoma cells resistant to ionizing radiation and chemotherapeutic agents that otherwise cause DNA damage [2-5]. Execution of cells by apoptosis usually requires the activation of cysteine proteases such as calpains and caspases [6]. Diverse stimuli may cause an increase in intracellular free [Ca^2+^], which is absolutely required for activation of calpain [7]. Activation of caspases may occur via different mechanisms [8,9]. Mitochondria mediated pathway of apoptosis may be activated in course of cell death. This involves the regulation of apoptosis by the Bcl-2 family proteins via controlling the release of cytochrome *c *from mitochondria [10,11], and subsequent formation of the cytosolic 'apoptosome' complex [12,13], which ultimately activates caspase-3 for execution of cells. Thus, the members of the Bcl-2 family modulate the mitochondrial pathway of apoptosis [14]. The pro-apoptotic (e.g., Bax, Bcl-xS) and anti-apoptotic (e.g., Bcl-2, Bcl-xL) members of this family, respectively, promote and inhibit the translocation of cytochrome *c *from mitochondria to cytosol [15].

Glucocorticoids are steroid hormones, which are secreted in response to stress and can modulate the ability of cells to undergo apoptosis [16]. For example, glucocorticoids induce apoptosis in thymocytes [17] and also increase the sensitivity of hippocampal neurons to cell death [18]. In contrast, DXM has been reported to induce resistance to certain drugs in glioblastoma cell lines [3-5]. Although an association with p21^WAF1/CIP1 ^protein accumulation has been reported [19], the exact mechanism of DXM mediated protection of glioblastoma cells from apoptosis is still largely unclarified. Exposure of human astrocytoma D384 and rat glioblastoma C6 cells to staurosporine induced apoptosis but pretreatment of those cells with DXM caused reduction in staurosporine mediated apoptosis [20]. In addition, DXM also conferred protection against the induction of apoptosis by anti-cancer agents including camtothecin and etoposide [20]. It has also been shown that exposure of glioblastoma cells to glucocorticoids induces partial resistance to anti-cancer agents such as cisplatinum, methotrexate, vincristine, cytarabine, adriamycin, and teniposide [3-5]. DXM appears to interfere with p53-dependent pathways of drug toxicity since the glioblastoma cell lines (LN-229 and U87MG) with wild-type p53 status were protected from drug toxicity by DXM to a greater extent than the cell lines (LN-18, LN-308, and T98G) with mutant p53 [3-5]. It has been reported earlier that DXM mediated protection from cancer chemotherapy occurs via a p53-independent pathway of regulating p21^WAF1/CIP1 ^expression in glioblastoma cells but this effect appears to be cell-type specific [19]. Thus, there remains a concern of modulatory effects of DXM on the mechanism of action of any chemotherapeutic agent for treatment of glioblastoma. Therefore, we have initiated this investigation to examine the modulatory effect of DXM on temozolomide (TMZ) induced apoptosis of glioblastoma cells. In an in vitro model using the human glioblastoma U87MG cells, we have investigated whether DXM confers resistance to TMZ action via inhibition of apoptosis.

TMZ is an alkylating chemotherapeutic drug that readily crosses the blood-brain-barrier in glioblastoma patients [21]. It is chemically related to decarbazine and is the 3-methyl derivative of the experimental anti-cancer drug mitozolomide. It has shown anti-tumor activity and relatively low toxicity in Phase I and Phase II clinical trials in patients with various advanced cancers, including malignant glioblastomas [21]. TMZ is spontaneously hydrolyzed under physiological conditions to its active metabolite 5-(3-methyltriazen-1-yl) imidazole-4-carboxamide (MTIC) [22]. The mechanism of action of MTIC is proposed to be methylation of DNA at the O_6 _position of guanine, with an additional methylation at its N_7 _position [23,24]. However, O_6_-methylguanine (O_6_-meG) may be removed by O_6_-methylguanine methyl transferase (MGMT) [25]. Cells deficient in MGMT do not repair O_6_-meG. Replication of DNA introduces a T instead of C opposite to O_6_-meG, resulting in GT mismatches [26]. Activation of mismatch repair system (MMRS) may remove T during DNA repair synthesis. However, ineffective MMRS causes growth arrest and eventually apoptotic death [27]. These studies have helped define the action of TMZ in cancer cells of mostly hemopoietic origin. However, the action of TMZ in glioblastoma cells remains largely undefined. Glioblastomas are relatively resistant to anti-cancer agents that cause apoptosis via DNA alkylation. Therefore, we have investigated the mechanism of TMZ induced apoptosis in human glioblastoma U87MG cells.

We found that TMZ caused apoptosis in U87MG cells as detected by morphological and biochemical assays. Alterations in the levels of pro-apoptotic Bax and anti-apoptotic Bcl-2 proteins are known to regulate the commitment to apoptosis [14,15]. Therefore, we investigated the levels of these apoptosis regulatory proteins following treatment of U87MG cells with TMZ. The initiation of apoptosis in U87MG cells following exposure to TMZ requires activation of calpain, a Ca^2+^-dependent cysteine protease, which plays a role in the mechanism of cell death in human malignant brain tumors including glioblastoma [28]. Besides, caspase-3 activity was also increased in TMZ induced apoptosis in U87MG cells. Pretreatment of U87MG cells with DXM blocked TMZ induced apoptosis, indicating that DXM worked as an antagonistic agent in TMZ induced apoptosis in human glioblastoma cells. The knowledge gained from our investigation implies that the combination of DXM and TMZ for the treatment of human glioblastoma patient may result in an undesirable clinical outcome. Preliminary results of this investigation have previously been presented [29].

## Results

### Evaluation of viability and apoptotic death both morphologically and biochemically

**Figure 1:**
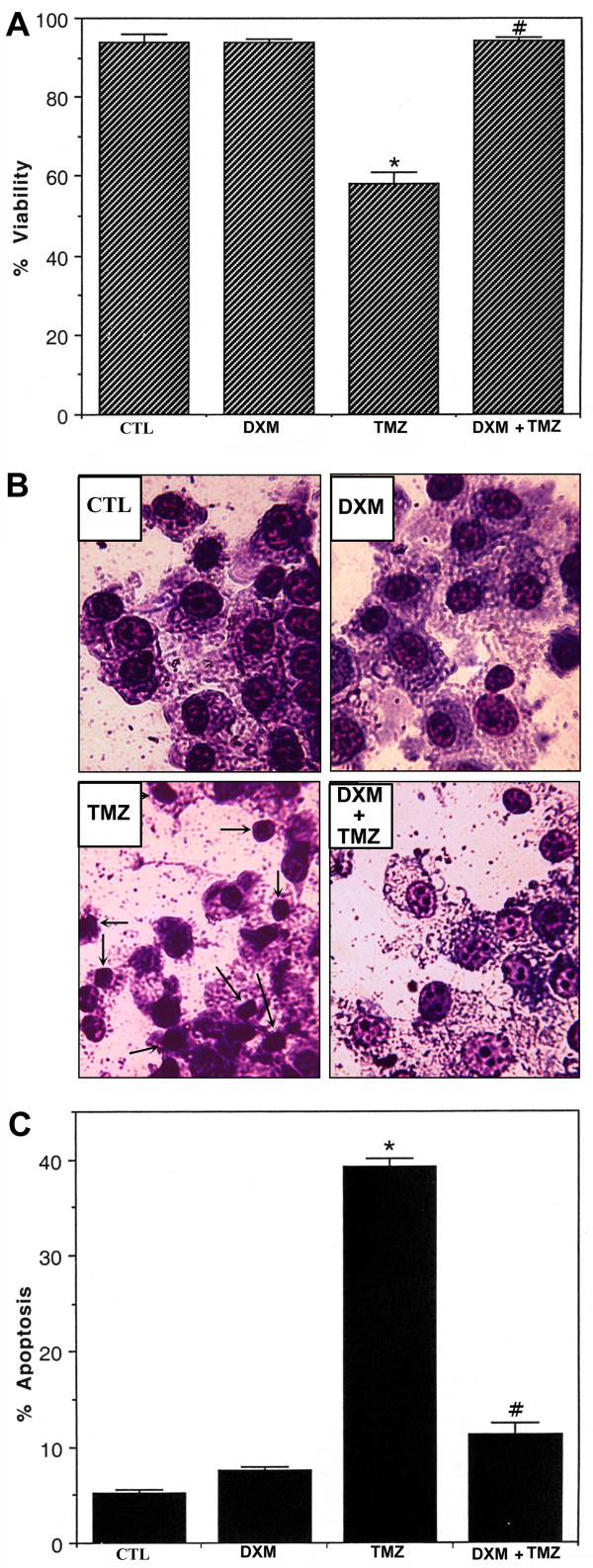
**Determination of apoptosis based on morphological features. **Four treatment groups: control (CTL); 40 μM dexamethasone (DXM) for 8 h; 100 μM temozolomide (TMZ) for 6 h; pretreatment with 40 μM DXM for 2 h followed by 100 μM TMZ for 6 h. (A) DXM prevented TMZ mediated decrease in U87MG cell viability. The trypan blue exclusion assay was used to assess cell viability in U87MG cells. (B) Photomicrographs showing representative cells from each treatment group. The arrows indicate apoptotic cells. (C) Bar graphs indicating the percentage of apoptotic cells counted from each group. Significant difference between CTL and TMZ treated cells was indicated by * (*P *≤ 0.05) and significant difference between TMZ treated cells and DXM plus TMZ treated cells was indicated by # (*P *≤ 0.05).

Exclusion of trypan blue dye by viable U87MG cells was evaluated under a light microscope using a hemocytometer after all treatments. A pretreatment with DXM prevented decrease in cell viability (panel A, Fig. 1). Morphological features of apoptosis were detected following Wright staining (panel B, Fig. 1) and counted to determine the amount of apoptotic cell death (panel C, Fig. 1) based on characteristic morphological features such as condensation of the nucleus and cytoplasm, cytoplasmic blebbing, and the formation of apoptotic bodies. All treatment groups were examined under the light microscopy and cells were counted to determine the percentage of apoptotic cells (panel C, Fig. 1). Compared to control (CTL) cells, cells treated with 100 μM TMZ showed an increase in the percentage of apoptotic cells (*P *< 0.001). A pretreatment of cells with 40 μM DXM decreased TMZ induced apoptosis by three-fold, compared to treatment of cells with TMZ only.

**Figure 2:**
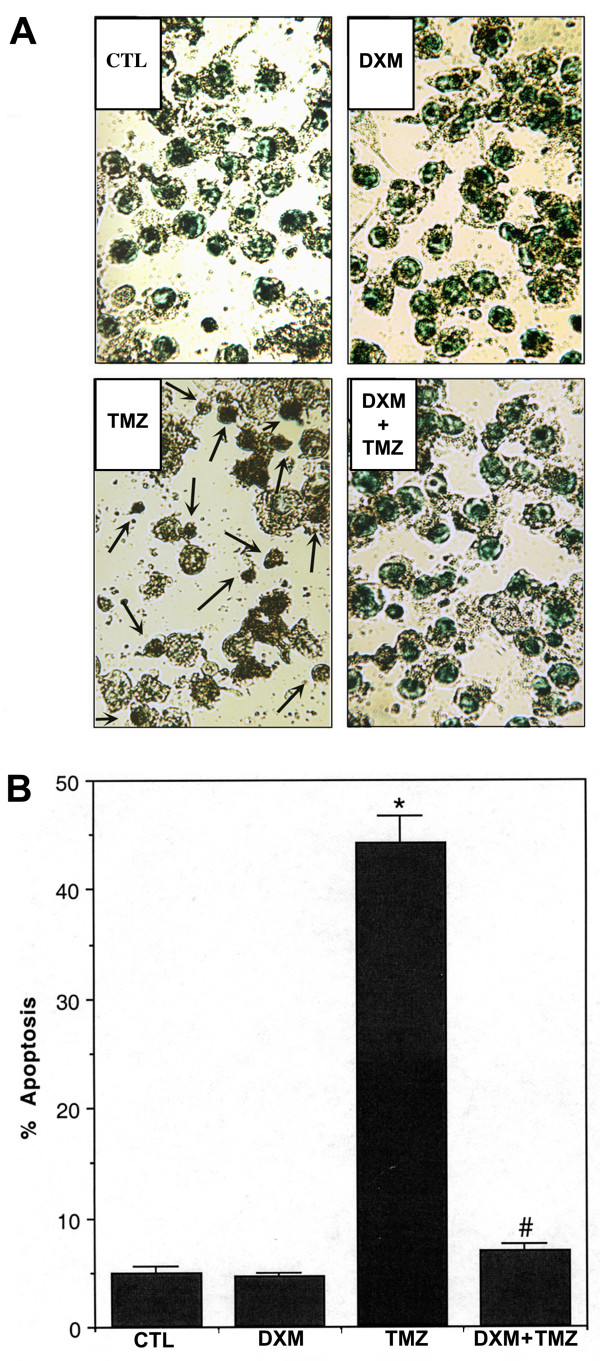
**ApopTag assay for detection and determination of DNA fragmentation in U87MG cells. **Four treatment groups: control (CTL); 40 μM dexamethasone (DXM) for 8 h; 100 μM temozolomide (TMZ) for 6 h; pretreatment with 40 μM DXM for 2 h followed by 100 μM TMZ for 6 h. (A) The photomicrographs showing representative cells from each treatment group. The arrows indicate apoptotic cells. (B) Bar graphs indicating the average percentage of apoptotic cells counted from each group. Significant difference between CTL and TMZ treated cells was indicated by * (*P *≤ 0.05) and significant difference between TMZ treated cells and DXM plus TMZ treated cells was indicated by # (*P *≤ 0.05).

Results obtained from Wright staining were further supported by the ApopTag assay (panel A, Fig. 2). Both CTL and DXM treated cells showed little or no brown color, confirming almost absence of ApopTag positive cells or apoptosis. The percentage of ApopTag positive cells was calculated (panel B, Fig. 2) and found to be highly significant (*P *< 0.001) in TMZ treated cells, compared to CTL cells. A pretreatment of cells with DXM considerably attenuated apoptotic DNA fragmentation in TMZ treated cells.

### Treatment with TMZ increased intracellular free [Ca^2+^]

**Figure 3:**
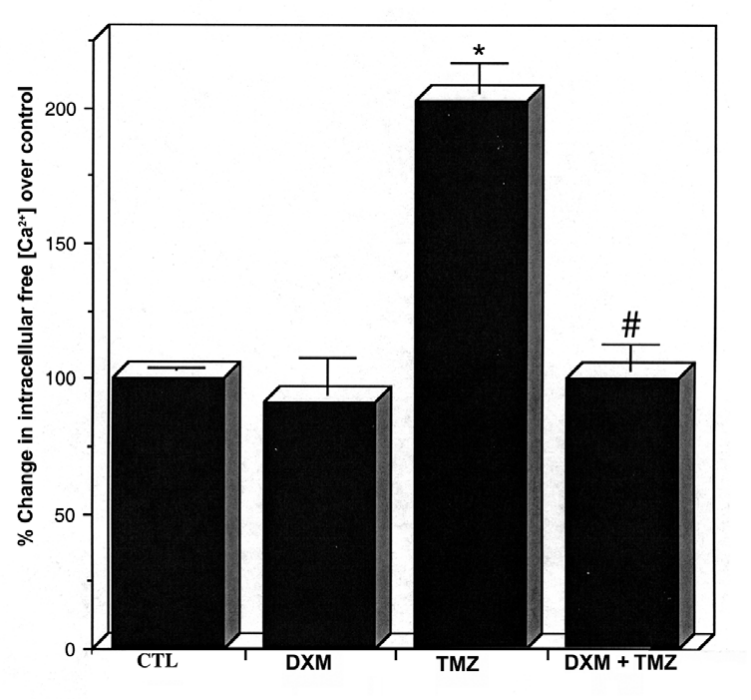
**Bar graphs indicating percentage of increase of intracellular free [Ca^2+^] using fura-2. **These data were generated from U87MG cells grown in phenol red-free medium for 24 h prior to treatments with the drugs in freshly prepared phenol red-free medium. Four treatment groups: control (CTL); 40 μM dexamethasone (DXM) for 8 h; 100 μM temozolomide (TMZ) for 6 h; pretreatment with 40 μM DXM for 2 h followed by 100 μM TMZ for 6 h. Percent changes in intracellular free [Ca^2+^] were shown at nM levels. Significant difference between CTL and TMZ treated cells was indicated by * (*P *≤ 0.05) and significant difference between TMZ treated cells and DXM plus TMZ treated cells was indicated by # (*P *≤ 0.05).

Using fura-2 assay, intracellular free [Ca^2+^] was determined in all treatment groups (Fig. 3). No significant difference (*P *= 0.928) was seen between CTL cells and cells treated with DXM alone. Cells treated with TMZ showed a significant increase (*P *= 0.007) in intracellular free [Ca^2+^], compared to CTL cells. This increase was attenuated almost 100% by a pretreatment of the cells with DXM. There was no significant difference (*P *= 0.999) between intracellular free [Ca^2+^] in CTL cells and those treated with DXM plus TMZ.

### TMZ induced apoptosis with an increase in Bax:Bcl-2 ratio

**Figure 4:**
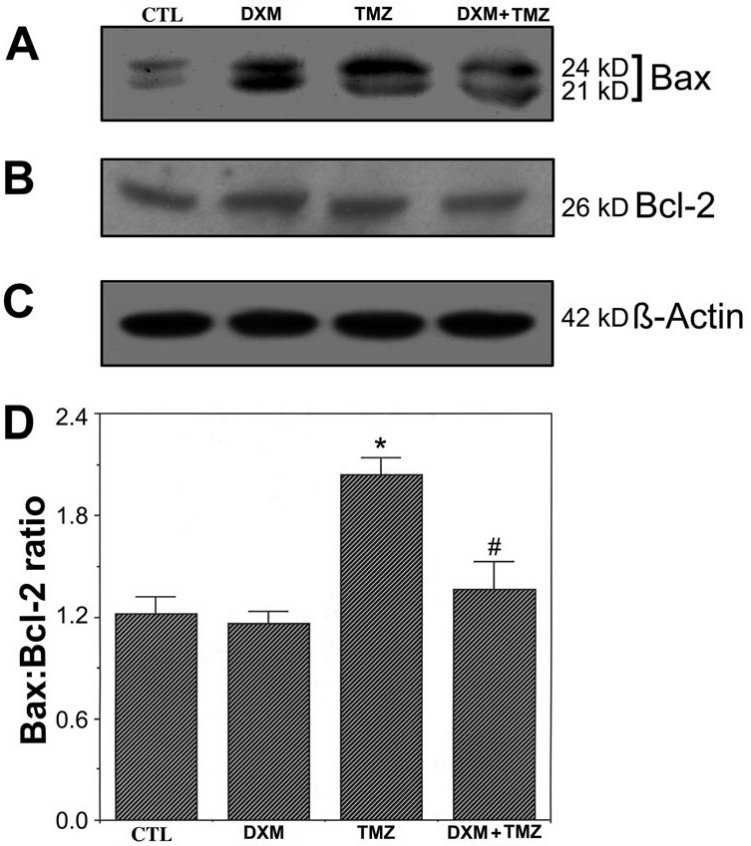
**The Bax:Bcl-2 ratio measured by Western blot analysis. **Four treatment groups: control (CTL); 40 μM dexamethasone (DXM) for 8 h; 100 μM temozolomide (TMZ) for 6 h; pretreatment with 40 μM DXM for 2 h followed by 100 μM TMZ for 6 h. (A) A representative gel picture showing level of expression of Bax. (B) A representative gel picture showing level of expression of Bcl-2. (C) A representative gel picture showing level of expression of β-actin. (D) Densitometric analysis showing the Bax:Bcl-2 ratio in all treatment groups. Significant difference between CTL and TMZ treated cells was indicated by * (*P *≤ 0.05) and significant difference between TMZ treated cells and DXM plus TMZ treated cells was indicated by # (*P *≤ 0.05).

A commitment to apoptosis was measured by examining any increase in the ratio of Bax (pro-apoptotic protein) expression to Bcl-2 (anti-apoptotic protein) expression. The bax gene encodes different isoforms. The antibody we used in this investigation could recognize 21 kD Baxα and 24 kD Baxβ bands (panel A, Fig. 4). Here, we considered both bands in our estimation of total Bax expression. We also examined the level of Bcl-2 expression in all treatment groups (panel B, Fig. 4). Almost same level of β-actin expression in each treatment ensured that equal amount of protein was loaded in each lane (panel C, Fig. 4). Based on the Western blot experiments (panels A and B, Fig. 4), the Bax:Bcl-2 ratios were measured in all treatment groups (panel D, Fig. 4). There was no significant difference (*P *= 0.983) in Bax:Bcl-2 ratio between CTL and DXM treated cells (panel D, Fig. 4). Compared to CTL cells, a rise in Bax:Bcl-2 ratio (panel D, Fig. 4) in cells exposed to TMZ was influenced more by a change in Bax expression (panel A, Fig. 4) than a change in Bcl-2 expression (panel B, Fig. 4). Compared to CTL cells, cells treated with TMZ showed a significant increase (*P *= 0.007) in the Bax:Bcl-2 ratio (panel D, Fig. 4). There was a significant difference (*P *= 0.019) in Bax:Bcl-2 ratio between cells treated with TMZ alone and those treated with DXM plus TMZ, indicating a loss of commitment to apoptosis due to a pretreatment with DXM. There was no significant difference (*P *= 0.871) in Bax:Bcl-2 ratio between CTL cells and cells pretreated with DXM and then treated with TMZ.

### Calpain and caspase-3 activities as determined by α-spectrin degradation

**Figure 5:**
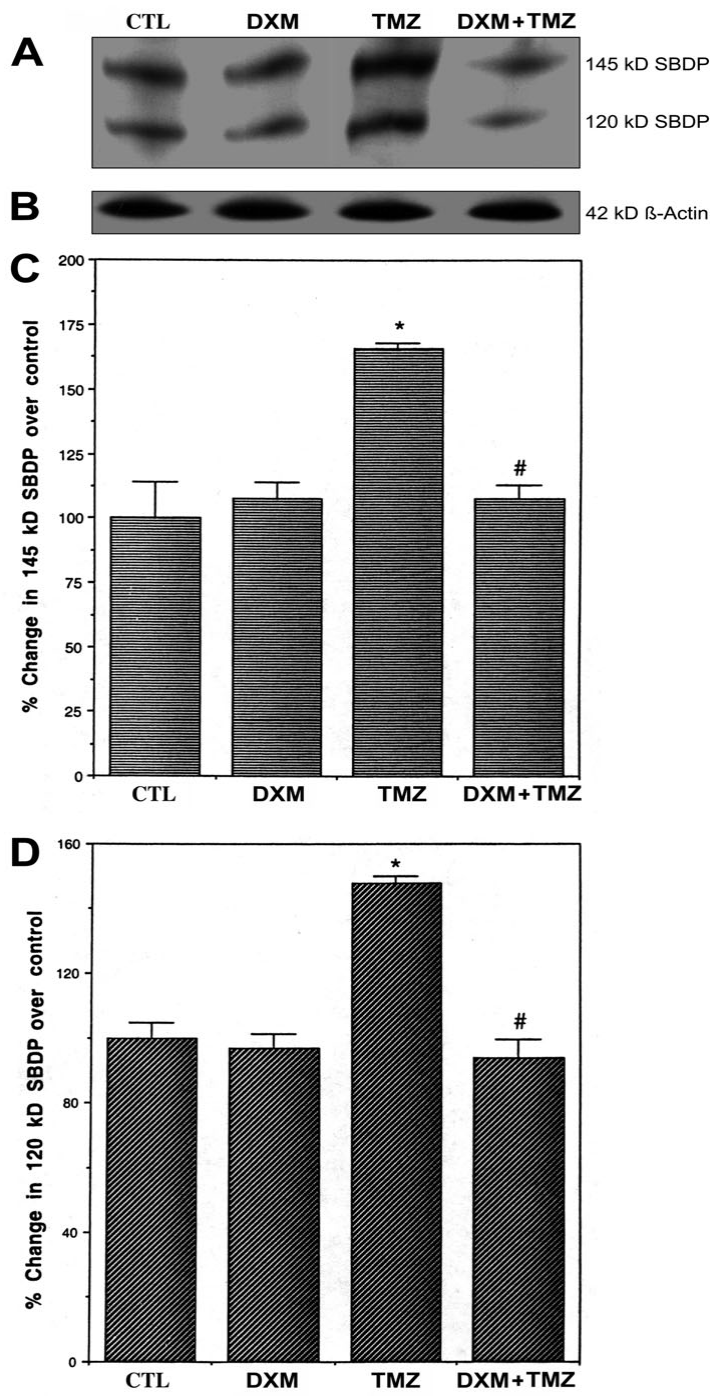
**Determination of calpain and caspase-3 activities using Western blot analysis of α-spectrin breakdown products (SBDPs). **Four treatment groups: control (CTL); 40 μM dexamethasone (DXM) for 8 h; 100 μM temozolomide (TMZ) for 6 h; pretreatment with 40 μM DXM for 2 h followed by 100 μM TMZ for 6 h. (A) A representative gel picture showing generation of 145 kD and 120 kD SBDPs. (B) A representative gel picture showing level of expression of β-actin. (C) Densitometric analysis showing percent changes in optical density of the calpain-specific 145 kD SBDP over CTL. (D) Densitometric analysis showing percent change of optical density of the caspase-3-specific 120 kD SBDP over CTL. Significant difference between CTL and TMZ treated cells was indicated by * (*P *≤ 0.05) and significant difference between TMZ treated cells and DXM plus TMZ treated cells was indicated by # (*P *≤ 0.05).

Calpain and caspase-3 activities were assessed by Western blot analysis of the calpain-specific 145 kD SBDP and the caspase-3-specific 120 kD SBDP, respectively (panel A, Fig. 5). Level of β-actin expression, which was almost uniform in all treatments, was used as a loading control (panel B, Fig. 5). There was no significant difference (*P *= 0.911) between CTL cells and DXM treated cells in generation of 145 kD SBDP, indicating similar levels of calpain activity in these two cases (panel C, Fig. 5). The generation of 145 kD SBDP in cells treated with TMZ was about 1.5-fold more intense (*P *= 0.003) than CTL cells, indicating that the level of calpain activity was increased in cells due to treatment with TMZ. Cells pretreated with DXM and then treated with TMZ showed a significant decrease in the generation of 145 kD SBDP, indicating an inhibitory effect of DXM on TMZ mediated increase in calpain activity (panel C, Fig. 5).

Caspase-3 activity was also measured by Western blot analysis in the generation of caspase-3-specific 120 kD SBDP (panel D, Fig. 5). Compared to CTL cells, treatment of cells with DXM alone did not cause a significant change (*P *= 0.983) in caspase-3 activity. Caspase-3 activity in cells treated with TMZ was almost 1.5 times more (*P *= 0.001) than CTL cells (panel D, Fig. 5). Thus, pretreatment of cells with DXM prior to treatment with TMZ appeared to decrease the upregulation of caspase-3 activity. Furthermore, there was no significant difference (*P *= 0.785) between CTL cells and cells that were pretreated with DXM and then treated with TMZ (panel D, Fig. 5).

### Caspase-3 activation as determined by generation of caspase-3-p20 fragment

**Figure 6:**
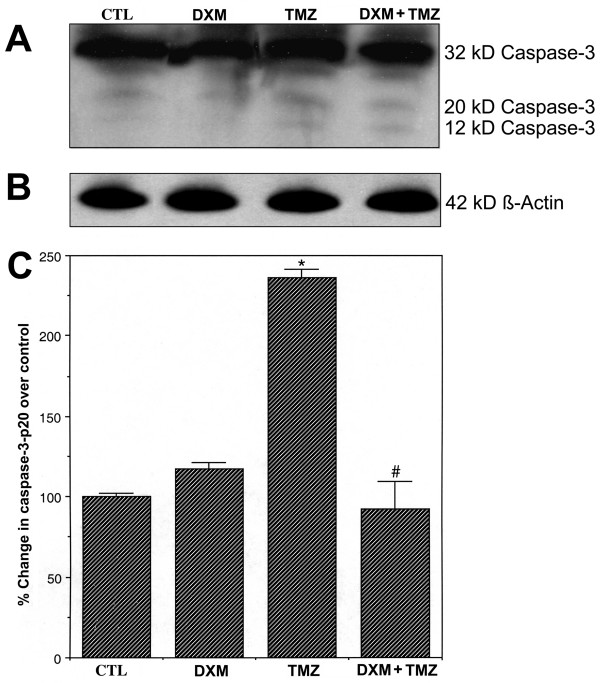
**Determination of caspase-3 activation using Western blot analysis of caspase-3-p20 active band. **Four treatment groups: CTL; 40 μM DXM for 8 h; 100 μM TMZ for 6 h; pretreatment with 40 μM DXM for 2 h followed by 100 μM TMZ for 6 h. (A) A representative gel picture showing caspase-3 activation. (B) A representative gel picture showing level of expression of β-actin. (C) Densitometric analysis showing percent change in optical density of the caspase-3-p20 active band over CTL. Significant difference between CTL and TMZ treated cells was indicated by * (*P *≤ 0.05) and significant difference between TMZ treated cells and DXM plus TMZ treated cells was indicated by # (*P *≤ 0.05).

Caspase-3 activation was also measured by Western blot analysis of the production of active 20 kD caspase-3 fragment (panel A, Fig. 6). Again, almost uniform expression of β-actin in all treatments served as an internal standard and indicated equal amounts of protein loadings in all lanes (panel B, Fig. 6). The intensities of active 20 kD caspase-3 band were almost similar in CTL cells and cells treated with DXM alone (panel C, Fig. 6). Treatment of cells with DXM alone did not cause a significant change (*P *= 0.613) in caspase-3 activation over CTL cells. But there was a significant increase (*P *= 0.001) in production of active 20 kD caspase-3 fragment in cells treated with TMZ, compared to CTL cells. Treatment of cells with DXM prior to TMZ appeared to significantly decrease the activation of caspase-3 (panel C, Fig. 6), indicating an inhibitory effect of DXM on TMZ induced caspase-3 activation.

## Discussion

Our studies indicated that pretreatment of human glioblastoma U87MG cells with DXM did not support chemotherapeutic action of TMZ. Treatment of U87MG cells with TMZ induced apoptosis (Figs. 1 and 2) to a significant extent by increasing intracellular free [Ca^2+^] (Fig. 3), interfering with the expression of apoptosis regulatory proteins of the Bcl-2 family resulting in upregulation of Bax:Bcl-2 ratio (Fig. 4), and increasing the activities of calpain and caspase-3 (Figs. 5 and 6). But a pretreatment of the cells with DXM prevented all these pro-apoptotic mechanisms (Figs. 3, 4, 5, 6). Our data also suggested that pretreatment with DXM can play a critical role in inhibiting Ca^2+ ^influx into the cells due to treatment with TMZ, and thus preventing the progression of apoptotic process.

Several in vitro studies documented a role for calpain in apoptosis of neuronal [30,31] as well as non-neuronal cells [32]. However, the mechanisms of calpain mediated cell death are not yet fully understood. Pro-apoptotic Bax is translocated to mitochondria and has shown to be activated by calpain [33]. Increased expression of calpain concurs with elevated expression of Bax relative to Bcl-2, suggesting that calpain overexpression plays an important role during cell death [34,35]. Because changes in expression of pro-apoptotic Bax and anti-apoptotic Bcl-2 control the mitochondrial pathway of apoptosis [14,15], we examined the levels of expression of Bax and Bcl-2 proteins in U87MG cells following treatment with TMZ (Fig. 4).

Our findings support a relationship between an increase in intracellular free [Ca^2+^] (Fig. 3) and cell death with an elevation of calpain activity (Fig. 5) following exposure of U87MG cells to TMZ. Pretreatment of cells with DXM showed a significant decrease in both intracellular free [Ca^2+^] and calpain activity in a subsequent exposure to TMZ. Increased intracellular free [Ca^2+^] causes activation of calpain and degradation of cytoskeletal proteins [36] with destabilization of the cellular integrity leading to cell death [37]. Our results indicate that DXM plays an important role in the prevention of calpain activation following treatment of glioblastoma cells with TMZ. DXM has been shown to inhibit apoptosis by induction of transcriptional expression of anti-apoptotic proteins Bcl-2 and Bcl-xL [37,38]. Upregulation of Bcl-2 either directly or indirectly can repress the Ca^2+ ^flux across the membrane of endoplasmic reticulum, thereby abrogating apoptosis via Ca^2+ ^signaling [39].

Treatment of U87MG cells with TMZ caused increase in calpain and caspase-3 activities as evidenced from the cleavage of α-spectrin at specific sites generating 145 kD SBDP and 120 kD SBDP, respectively (Fig 5). A pretreatment with DXM decreased calpain and caspase-3 activities in U87MG cells. Overall, the results from this investigation showed that DXM pretreatment interfered with proteolytic activities and apoptotic death in U87MG cells exposed to TMZ. A previous report from our laboratory indicated that corticosteroids could inhibit the proteolytic activity of calpain [40].

Our study suggests that pretreatment of glioblastoma with DXM should be avoided if there is a plan to treat the glioblastoma patients subsequently with TMZ. Some recurrent glioblastomas remain resistant to almost all current therapeutic endeavors, with low response rates and survival rarely exceeding six months. As there are no clearly established chemotherapeutic regimens for drug resistant glioblastomas, obviously the only aim of therapy is palliation with improvement in the quality of life. In such cases, use of DXM or other glucocorticoids may not be controversial. However, promising therapeutic activity of TMZ against newly diagnosed anaplastic astrocytomas and glioblastomas warrants continued evaluation of this agent in combination settings [41]. Delaying disease progression by treatment with TMZ is beneficial to the patients with recurrent glioblastomas [42]. Therefore, the use of this drug should be explored further in an adjuvant setting and in combination with other agents [43].

We showed that a pretreatment of human glioblastoma U87MG cells with a low dose of DXM abolished the chemotherapeutic action of TMZ (Figs. 1, 2, 3, 4, 5, 6), raising a renewed concern about the validity of DXM as a supportive therapy in the treatment of glioblastomas. We acknowledge that pharmacological studies with a glioblastoma cell line may not always yield results that are easily transferred to the in vivo situation for cancer therapy. Also, clinical recommendations should not be based on in vitro data alone. Nevertheless, our data strongly suggested that DXM treatment could well interfere with therapeutic efficacy of chemotherapy in human glioblastoma patients. In fact, this hypothesis is in line with the results from a 1983 clinical trial where the combination of bis(chloroethyl) nitrosourea (BCNU) plus high dose methylprednisolone, a steroid, tended to be less effective than BCNU alone in patients with poor prognosis [44].

The data reported here and the previous reports by others [45], taken together, provide enough reason to call for steroid withdrawal during investigative clinical trials of chemotherapeutic agents in glioblastoma patients. This should be a serious concern at the initial clinical situation when glioblastoma patients are enrolled. In the case of tumor progression during chemotherapy, steroids may still be life-saving agents, and individual decisions concerning a continuation of chemotherapy with concurrent steroid treatment must be made. We also suggest limiting steroid treatment in glioblastoma patients who are receiving chemotherapy outside a controlled clinical trial, because the benefit of chemotherapy for glioblastoma patients is still with limited efficacy and should not be further compromised by co-medication with steroid. Further investigations in xenografted and allografted animal models of glioblastoma as well as in human glioblastoma patients may shed new light in the controversial use of DXM in palliation of human glioblastoma patients.

## Materials and methods

### Cell culture and treatments

Human glioblastoma U87MG cells were purchased from the American Type Culture Collection (Manassas, VA). Cells were grown in 75-cm^2 ^flasks containing 10 ml of 1 × RPMI 1640 supplemented with 10% fetal bovine serum (FBS) and 1% penicillin and streptomycin in a fully-humidified incubator containing 5% CO_2 _at 37°C. Prior to drug treatments, the cells were starved in 1 × RPMI 1640 supplemented with 0.5% FBS for 24 h. Dose-response studies were conducted to determine the suitable doses of the drugs for using in the experiments. Cells were pretreated with 40 μM DXM for 1 h. The DXM pretreated or untreated cells were subsequently treated with 100 μM TMZ for 6 h. Cells were washed with drug-free medium and allowed to grow for 48 h. Then, cells were collected for determination of viability, apoptosis, or Western blot analysis. DXM and TMZ were obtained from Sigma Chemical (St. Louis, MO) and Schering Corporation (Kenilworth, NJ), respectively. The drugs were dissolved in dimethyl sulfoxide (DMSO) to make stock solutions, which were then stored at -20°C until used for treating cells.

### Trypan blue dye exclusion test for cell viability

Following all treatments the viability of attached and detached cell populations was evaluated by trypan blue dye exclusion test [46]. Viable cells maintained membrane integrity and did not take up trypan blue. Cells with compromised cell membranes took up trypan blue, and were counted as dead. At least 800 cells were counted in four different fields and the number of viable cells was calculated as percentage of the total cell population.

### Wright staining for morphological analysis of apoptosis

The cells from each treatment were washed with PBS, pH 7.4, and sedimented onto the microscopic slides using Cytobucket and Centra CL2 centrifuge (IEC) at 1200 rpm for 5 min. Cells were fixed in 95% (v/v) ethanol before examination of morphology with Wright staining [46]. The morphology of the apoptotic cells as detected by light microscopy included such characteristic features as chromatin condensation, cell-volume shrinkage, and membrane-bound apoptotic bodies. Four randomly selected fields were counted for at least 800 cells. The percentage of apoptotic cells was calculated from three separate experiments.

### ApopTag assay for biochemical detection of apoptotic DNA fragmentation

ApopTag Peroxidase kit (Intergen, Purchase, NY) was used to assess the extent of cell death following drug treatments. Briefly, cells for each treatment were grown on six-well cell culture plates (Corning Corporation., Corning, NY) and were treated as described above. Following treatments, cells were washed with PBS and then centrifuged to sediment onto the microscopic slides. Residual PBS was then removed and cells were fixed using 95% (v/v) ethanol and allowed to dry overnight. Slides were pretreated with a protein-digesting enzyme for 15 min and then washed with distilled water for 2 min. Cells were quenched with 3% (v/v) hydrogen peroxide for 5 min followed by washing with PBS. Terminal deoxynucleotidyl transferase (TdT) enzyme was added to the pre-equilibrated cells and incubated for 1 h at 37°C. Stop-buffer was added to the slide and agitated for 15 sec followed by 10 min incubation at room temperature. After washing three times with PBS for 1 min each, anti-digoxigenin peroxidase conjugate was added to the slides and incubated for 30 min. After slides were washed twice with PBS, freshly prepared peroxidase substrate 3,3'-diaminobenzidine was added to the slides and kept for 6 min and then slides were washed with water two times. Slides were counterstained with 0.5% (w/v) methyl green for 10 min followed by washing with water and then 100% n-butanol. After 10 min, cells were dehydrated in xylene for 2 min and then mounted with glass coverslip. Experiments were conducted in triplicates and the percentage of ApopTag-positive cells was determined by counting cells under light microscopy.

### Determination of intracellular free [Ca^2+^] using fura-2

We recently reported this method [47], which was modified for determination of intracellular free [Ca^2+^] in U87MG cells. Briefly, cells were grown to 80% confluency in phenol red-free medium for 72 h, suspended in the culture medium, centrifuged at 2000 rpm for 5 min to obtain pellet, and washed twice in phosphate buffered saline (PBS, pH 7.4). Cells were resuspended in culture medium, and incubated at 37°C for 2 h with gentle shaking. Following incubation, cells wee washed twice in Ca^2+^-free Locke's buffer [48] and then counted on a hemocytometer. Cells (2 × 10^7 ^cells/ml) were dispersed in Locke's buffer with 10% FBS. Fura-2 (Molecular Probes, Eugene, OR) was dissolved in DMSO and diluted in Ca^2+^-free Locke's buffer containing 10% FBS. Cells were mixed with 5 μM fura-2, incubated at 37°C for 30 min, washed twice and diluted to 1 × 10^6 ^cells/ml in Ca^2+^-free Locke's buffer. The intracellular free [Ca^2+^] was calculated spectrofluorometrically using the equation [Ca^2+^] = *K_d_β(R-R_min_)/(R_max_-R)*, where *β *is the ratio of F^380^_max_, fluorescence intensity exciting at 380 nM for zero free Ca^2+ ^to F^380^_min_, and fluorescence intensity at saturating free [Ca^2+^] as reported previously [49]. The determination of fluorescence ratio *(R) *was performed using an SLM 8000 fluorometer (Thermospectronic, Shelton, CT) at 340 and 380 nm wavelengths. The maximal *(R_max_) *and minimal *(R_min_) *ratios were determined using 200 μl of 250 μM digitonin (Sigma) and 500 mM EGTA (Sigma, St. Louis, MO), respectively. The value of *K*_*d*_, a cell-specific constant, was determined experimentally to be 0.476 μM using standards of the Calcium Calibration Buffer Kit with Magnesium (Molecular Probes, Eugene, OR).

### Antibodies

Monoclonal antibody against α-spectrin (Affiniti, Exeter, UK) was used to measure calpain activity as well as caspase-3 activity. Caspase-3 polyclonal antibody (MBL International, Woburn, MA) was used to determine caspase-3 activation. Bax and Bcl-2 monoclonal antibodies (Santa Cruz Biotechnology, Santa Cruz, CA) were used to assess apoptotic threshold by determining the Bax:Bcl-2 ratio. Antibody to β-actin (monoclonal clone AC-15, Sigma) was used to standardize protein loading in Western blot experiments. The secondary antibody was horseradish peroxidase (HRP)-conjugated goat anti-mouse IgG (ICN Biomedicals, Aurora, OH), except in case of calpain and α-spectrin where HRP-conjugated goat anti-rabbit IgG was used (ICN Biomedicals).

### Western blotting and ECL detection

Cells were washed in culture flasks using Hank's balanced salt solution without Ca^2+ ^(GIBCO, Grand Island, NY). The cells were then washed twice in phosphate buffered saline (PBS, pH 7.4) and centrifuged in Eppendorf 5804R (Brinkmann Instruments, Westbury, NY) at 106 × g for 10 min. Cells were resuspended in a homogenizing buffer composed of 50 mM Tris-HCl (pH 7.4), 1 mM PMSF (Bethesda Research Laboratories, Gaithersburg, MD), and 5 mM EGTA (Sigma). A polypropylene pestle (Kontes Glass, Vineland, NJ) was placed inside the microcentrifuge tube (1.5 ml) containing cells and the tube was placed inside an ice bucket for 2 min to let the pestle cool to 4°C. Cells were completely homogenized for 30 sec (the homogenization was performed while holding the microcentrifuge tube in ice with one hand). Following homogenization, protein concentration was determined using Coomassie Plus Protein Assay Reagent (Pierce, Rockford, IL) and spectrophotometric measurement at 595 nm (Spectronic, Rochester, NY). Samples were then diluted (1:1) in sample buffer (62.5 mM, Tris pH 6.8, 2% SDS, 5 mM β-mercaptoethanol, 10% glycerol) and boiled for 5 min. Samples were then loaded onto the 4–20% gradient gels for electrophoresis at 200 V for 30 min (Bio-Rad, Hercules, CA). For detection of α-spectrin bands, a 5% gel was used for electrophoresis at 100 V for 2 h. Following electrophoresis, gels with the resolved proteins were electroblotted to nylon membranes (Millipore, Bedford, MA) in an electroblotting Genie apparatus (Idea Scientific, Minneapolis, MN). The membranes were blocked for 1 h in blocking buffer (5% powdered non-fat milk, 20 mM Tris pH 7.6, 0.1% Tween 20 in saline). Primary antibody was diluted (1:100 for Bax, Bcl-2, caspase-3, and 1:500 for calpain, 1:2,000 for α-spectrin, and 1:15,000 for β-actin) in blocking solution and then added to the blots for 1 h. The blots were washed three times with a wash buffer (Tris/Tween solution) and covered with secondary antibody (goat anti-rabbit for calpain and α-spectrin and goat anti-mouse for all others) at a 1:2000 dilution for 1 h. Blots were incubated with enhanced chemiluminescence (ECL) detection system (Amersham Pharmacia, Buckinghamshire, UK) and exposed to X-OMAT AR films (Eastman Kodak, Rochester, NY). The films were scanned on a UMAX PowerLook Scanner (UMAX Technologies, Fremont, CA) using Photoshop software (Adobe Systems, Seattle, WA), and optical density of each band was determined using Quantity One software (Bio-Rad, Hercules, CA). Estimation of the degradation products of α-spectrin indicated calpain and caspase-3 activities. The 145 kD spectrin breakdown product (SBDP) is specific for calpain activation [30], and the 120 kD SBDP is specific for caspase-3 activation [50]. Also, Western blot analysis was performed to examine the generation of active 20 kD caspase-3 fragment from 32 kD caspase-3, indicating activation of caspase-3.

### Statistical analysis

Data from various experiments were analyzed using StatView software (Abacus Concepts, Berkeley, CA). Results were compared using one-way analysis of variance (ANOVA) with Fisher's protected least significant difference (PLSD) post hoc test at a 95% confidence interval. All results were presented as mean ± standard error of mean (SEM) of separate experiments (n ≥ 3). A difference between two values was considered significant at *p *≤ 0.05.

## Authors' contributions

AD performed the experiments and participated in writing the manuscript. NLB contributed to the interpretation of results. SJP participated in the discussion of the study and provided comments on clinical importance of this study. SKR conceived the study, planned experimental design, supervised the study, and helped writing the manuscript. All authors read and approved the final version of the manuscript.
